# O08 A case of juvenile systemic lupus erythematosus with pyrexia of unknown origin

**DOI:** 10.1093/rap/rkab067.007

**Published:** 2021-10-19

**Authors:** Kishore Warrier, Afraa Al-Sabbagh, Samundeeswari Deepak

**Affiliations:** 1 Nottingham University Hospitals, Nottingham, United Kingdom; 2 Peterborough City Hospital, Peterborough, United Kingdom

## Abstract

**Case report - Introduction:**

An 18-year-old young man with diagnosis of Juvenile SLE with lupus nephritis maintained in remission on Mycophenolate Mofetil (MMF), presented with high-grade fever without focus close to his 18th birthday. History or examination did not point to any focus of infection, but blood tests showed systemic inflammation. Extensive investigations, including extended infection screen and bone marrow aspiration, were negative. His blood tests were suggestive of macrophage activation syndrome with no clinical or laboratory evidence of flare of lupus. MRI scan revealed extensive para-aortic lymphadenopathy and a diagnosis of classical Hodgkin's lymphoma confirmed on lymph node biopsy.

**Case report - Case description:**

Interestingly he first presented with fever and cervical lymphadenopathy at 13 years of age to local hospital, when a biopsy had revealed lymphoma but the diagnosis was changed to SLE following review at tertiary hospital. He was started on steroids and low-dose methotrexate. He later developed facial swelling, abdominal distension and groin swelling, following which he was referred to tertiary renal centre. His examination revealed scarring malar rash, anaemia, ankle and scrotal oedema, ascites and pleural effusion. His investigations showed anaemia, thrombocytopaenia, ESR over 100mm/hour, hypoalbuminaemia, low complement levels, positive ANA and double-stranded DNA. He had nephrotic range proteinuria and renal biopsy showed Class V lupus nephritis. He was treated with intravenous and later weaning dose of oral steroids and started on MMF. He remained in remission on a stable dose of MMF, except for brief flare of proteinuria, which settled with short course of oral steroids.

A few months before his eighteenth birthday, he presented to local hospital with recurrent episodes of high-grade fever with no clear focus. He was twice managed with broad spectrum antibiotics, despite negative septic screen and an extended infection screen including viral PCRs and IGRA for tuberculosis. He had no focal symptoms or symptoms to suggest flare of underlying lupus, but had loss of appetite and loss of weight. He developed splenomegaly with increasing inflammatory markers and fall in all cell lines of full blood count with deranged coagulation. He satisfied the clinical and laboratory criteria of the preliminary Diagnostic Guidelines for Macrophage Activation Syndrome in JSLE (2009), following which he was commenced on three days of IV methylprednisolone following normal bone marrow aspirate. However, fever recurred after initial response and MRI scan revealed mediastinal and para-aortic lymphadenopathy, and a diagnosis of classical Hodgkin's lymphoma was confirmed on lymph node biopsy.

**Case report - Discussion:**

This young man initially presented with fever and cervical lymphadenopathy, when a diagnosis of lymphoma was made. Yet, following review of the biopsy at the local tertiary centre and the abnormal immunological profile, a diagnosis of lupus was made. He then developed nephrotic syndrome, when a diagnosis of lupus nephritis was confirmed on biopsy. Other clinical and laboratory profile (raised ESR, hypocomplementeamia, serositis and haematological parameters) corroborated the diagnosis. He responded well to steroids and immunosuppression, when he presented with fever again. He had no evidence of flare of underlying lupus or any infection, but blood tests and splenomegaly pointed to macrophage activation syndrome (MAS). A negative bone marrow examination suggested that pancytopaenia was unlikely to be due to bone marrow infiltration, which is why he was treated with steroids. Yet, when fever recurred, MRI was arranged to investigate further, which eventually led to revelation of lymphadenopathy and biopsy, which eventually led to the right diagnosis. Another dilemma was if his MAS was to respond to steroids, whether we should change his baseline immunosuppression which had worked well so far for him and if we chose to, what would be the best agent.

**Case report - Key learning points:**

1. Although extremely uncommon, children and young people with rheumatic diseases can develop malignancy, which may be unrelated to the primary condition or the therapeutic agents used. Unexplained symptoms in the absence of flare of underlying condition and with infections ruled out, warrant extension of investigations to rule out an underlying malignancy.

2. Diagnosis of secondary MAS in a rheumatological condition without any evidence of flare of the underlying condition should be viewed as suspicious and warrants more investigations to rule out other causes.

3. In patients who develop MAS, there are very few agents which work for both lupus and MAS, choosing an agent which treats both conditions is challenging.

